# *M. tuberculosis* enhances its virulence during replication in blood from HIV patients

**DOI:** 10.1186/1471-2334-14-S3-O12

**Published:** 2014-05-27

**Authors:** Michelle B Ryndak, Krishna K Singh, Zhengyu Peng, Hualin Li, Lu Meng, Suman Laal

**Affiliations:** 1 Veterans Affairs Medical Center, New York Harbor Health Care System, New York, NY, USA; 2 Institutes of Biomedical Sciences, Shanghai Medical College, Fudan University, Shanghai, China; 3 Center for Virology and Vaccine Research, Beth Israel Deaconess Medical Center, Harvard Medical School, Boston, MA, USA; 4 New York University Langone Medical Center, Departments of Pathology and Microbiology, New York, NY, USA

## Background

*Mycobacterium tuberculosis* and HIV act synergistically to enhance and accelerate the development of tuberculosis and progression of HIV infection to AIDS. Hematogenous dissemination of *M. tuberculosis* leading to extrapulmonary TB, disseminated TB and miliary TB is greatly increased in HIV+ TB patients. We have compared the transcriptome of *M. tuberculosis* replicating in whole blood from immunocompetent and immunodeficient individuals to understand how *M. tuberculosis* adapts to the blood environment during hematogenous dissemination.

## Methodology

Whole genome microarray analysis was performed on RNA from *M. tuberculosis* replicating in whole blood from PPD negative HIV- healthy donors and HIV+ donors. Genes with a fold change of≥ 2, at a false discovery rate of < 2%, were considered significantly differentially expressed.

## Results

*M. tuberculosis* survives and replicates in blood, and enhances its virulence/pathogenic potential during adaptation to the hematogenous environment. The blood specific transcriptome reflects suppression of dormancy, induction of cell-wall remodeling, alteration in mode of iron acquisition, evasion of immune surveillance and enhanced expression of important virulence factors that drive active infection and dissemination. Compared to replication in HIV blood, these changes are accentuated during replication in blood from HIV patients. The expression of ESAT-6, known to play an important role in dissemination of *M. tuberculosis* from the lungs, is upregulated in *M. tuberculosis* growing in blood, and especially dramatically during growth in HIV+ blood.

## Conclusion

*M. tuberculosis* modulates its aggressive progression to disseminated forms of TB by modifying its transcriptome to acquire a phenotype with enhanced virulence that favors active infection and dissemination.

